# The Lancet Psychiatry Commission on Transforming Mental Health Implementation Research

**DOI:** 10.1192/j.eurpsy.2025.152

**Published:** 2025-08-26

**Authors:** E. McGinty

**Affiliations:** Health Policy & Economics, Weill Cornell Medicine, New York, United States

## Abstract

**Abstract:**

While many evidence-based mental health prevention, promotion, and treatment interventions exist, they are poorly and inequitably scaled across populations. Too often, research produces interventions and implementation strategies that are difficult to scale due to misalignment with culture, policy, system, community, provider, and individual realities. This presentation will introduce the Lancet Psychiatry Commission on Transforming Mental Health Implementation Research, which makes five recommendations to transform the research enterprise to produce more actionable evidence and address the mental health implementation gap. These recommendations focus on strategies for integrating research and real-world implementation; centering health equity in mental health intervention and implementation research; using a complexity science lens to study strategies for scaling effective interventions; expanding research designs beyond the traditional randomized clinical trial; and using transdisciplinary approaches. The Commission also made cross-cutting recommendations related to elevating work on mental health systems and policy and the importance of strengthening mental health implementation research globally.

**Disclosure of Interest:**

None Declared

